# Transposon age and non-CG methylation

**DOI:** 10.1038/s41467-020-14995-6

**Published:** 2020-03-06

**Authors:** Zhengming Wang, David C. Baulcombe

**Affiliations:** Department of Plant Sciences, University of Cambridge, Cambridge, CB2 3EA UK

**Keywords:** Gene silencing, Plant molecular biology

## Abstract

Silencing of transposable elements (TEs) is established by small RNA-directed DNA methylation (RdDM). Maintenance of silencing is then based on a combination of RdDM and RNA-independent mechanisms involving DNA methyltransferase MET1 and chromodomain DNA methyltransferases (CMTs). Involvement of RdDM, according to this model should decrease with TE age but here we show a different pattern in tomato and *Arabidopsis*. In these species the CMTs silence long terminal repeat (LTR) transposons in the distal chromatin that are younger than those affected by RdDM. To account for these findings we propose that, after establishment of primary RdDM as in the original model, there is an RNA-independent maintenance phase involving CMTs followed by secondary RdDM. This progression of epigenetic silencing in the gene-rich distal chromatin is likely to influence the transcriptome either in *cis* or in *trans* depending on whether the mechanisms are RNA-dependent or -independent.

## Abstract (web summary)

RNA-directed DNA methylation (RdDM) is thought to silence newly inserted transposable elements (TEs) with RNA-independent mechanisms becoming more prominent as TEs age. Here, the authors show that RdDM continues to silence the oldest intact distal TEs in tomato and Arabidopsis suggesting a second, later phase of RdDM.

## Introduction

Epigenetic control of genomes is associated with chemical modifications of DNA and histone^1^, or both, that are often linked to silencing of transposable elements (TEs) as part of a genome defense system^2^. There are also effects of these modifications on gene expression, chromosome behavior and differentiation of pericentric heterochromatin and distal euchromatin. Full understanding of genomes, therefore, requires knowledge of factors affecting the establishment and maintenance of epigenetic marks. The simplest explanation of these factors invoke DNA sequence elements or structures that are associated with recruitment of DNA- or histone-methyltransferases to chromatin domains through the RNA-directed DNA methylation (RdDM) and other pathways^2^. Once established, DNA methylation can be maintained through cell division by different factors depending on the sequence context^2,3^.

To investigate factors influencing epigenetic marks in plants we focus here on tomato in which there are large and well-separated pericentric and distal chromatin domains^4,5^. In *Arabidopsis*, in contrast, the pericentric region is relatively small and largely restricted to narrow domains around the centromere and small knobs on chromosome 4^6^. DNA methylation in tomato, as in other plants, occurs at CG, CHG and CHH sequence contexts (H indicates A, T, or C). Loss of CG methylation in the *met1* mutant disrupts growth rate, flowering time, and gametogenesis in *Arabidopsis*^7–9^. In contrast the loss of CHG or CHH methylation does not show any obvious phenotype in *Arabidopsis*^10^, presumably because the levels of CHG/CHH methylation in WT is lower than CG methylation^11,12^.

To gain insights into the roles of CHG methylation in tomato, we utilized CRISPR to knock out tomato homologs of KYP and CMT3 which are required for maintenance of CHG methylation^13,14^. Genome-wide methylation profiles reveal that, as in *Arabidopsis*, SlKYP and SlCMT3 are required for CHG methylation maintenance, and CHH methylation to a lesser extent. However, unlike *Arabidopsis*, single *slkyp* or *slcmt3* mutants have pleiotropic phenotypes affecting growth, development and reproduction. Our analysis of these mutants together with the *slnrpd1* mutant^15^ identifies anti-correlation between CMT3-mediated and RdDM-mediated TE silencing in distal chromatin. In terms of intact long terminal repeats (LTR) transposons, CMT3 tends to target younger elements than RdDM.

## Results

### CRISPR-based mutation of *SlKYP* and *SlCMT3*

Tomato is a good epigenetic model plant because it has a clearly delineated heterochromatic region in the pericentromere. There are mutants and knock down lines of epigenetic pathway genes and a well characterized effect of DNA methylation in fruit ripening^15–18^. To extend our previous analysis of DNA methylation in tomato we used CRISPR small guide RNAs (sgRNAs) to target tomato orthologs of *AtCMT3* and *AtKYP*—the major genes involved in CHG methylation in *Arabidopsis*^13,14^. AtKYP binds methylated CHG, mediates H3K9 di-methylation that, in turn, recruits CMT3^19,20^. The *SlMET1l* (Solyc01g006100) and *SlMET3l* (Solyc12g100330)^21,22^ loci are two tomato orthologs with similarity to *AtCMT3* (Supplementary Fig. 1a) and we refer to them as (*Sl)CMT3a* and (*Sl)CMT3b*. The tomato ortholog of *AtKYP* is *SlSDG5* (Solyc02g094520) (Supplementary Fig. 1b,c)^23^ and we refer to it as (*Sl)KYP*.

Consistent with the functions of their *Arabidopsis* orthologs the *CMT3a, CMT3b,* and *KYP* mutations (Supplementary Fig. 2) had no effect on CG methylation (Supplementary Figs. 3a and 4a)^24^. However, also as in *Arabidopsis*, CHG methylation in *cmt3a* and *kyp* was lower than WT with *kyp* having a stronger effect than *cmt3a* (Supplementary Fig. 3b). There was also a genome-wide reduction of CHH methylation in *kyp* (Supplementary Fig. 3c). In *cmt3b* there was no effect in any context (Supplementary Fig. 4). From these results we conclude that SlCMT3a rather than SlCMT3b is the primary functional homolog of AtCMT3. Most (89%) of the differentially methylated regions (DMRs) (CHG hypomethylation) in *kyp* (Supplementary Fig. 5a,b) overlapped with *cmt3a* DMRs (Supplementary Fig. 5c). The CHH *kyp* and *cmt3a* hypoDMRs represent subsets of *kyp* and *cmt3a* CHG hypoDMRs (Supplementary Fig. 5c).

**Fig. 1:**
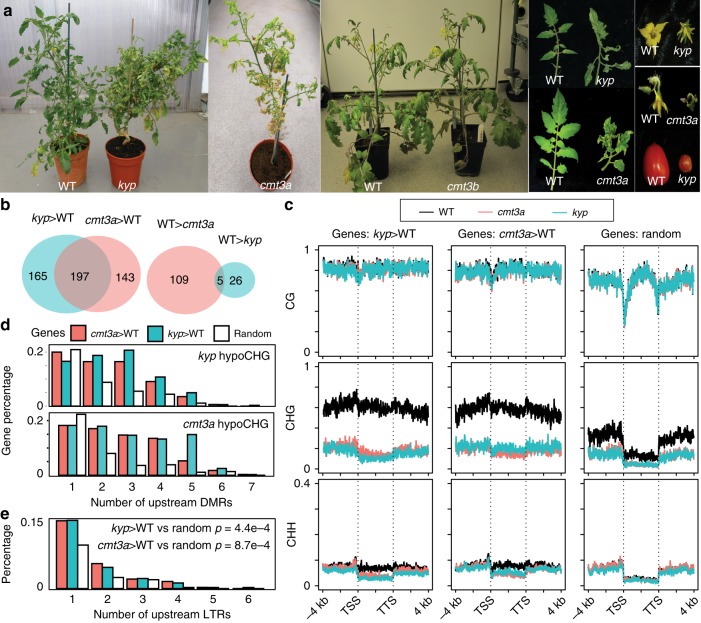
SlKYP and SlCMT3a are required for tomato growth, development and reproduction. **a** Mutants phenotypes. One-month-old plants, leaves from a single branch, flowers and fruits of WT and mutants were shown. **b** Venn diagrams showing overlap analysis of differentially expressed (DE) genes; *kyp* > WT, upregulated genes in *kyp*; *cmt3a* > WT, upregulated genes in *cmt3a*; WT > *cmt3a*, downregulated genes in *cmt3a*; WT > *kyp*, downregulated genes in *kyp*. The upregulated genes are not mis-annotated TEs because 271 of 340 *cmt3a*-upregulated genes and 289 of 362 *kyp*-upregulated genes are well annotated protein-coding sequences with high confidence (Supplementary Data 1 and 2). **c** Average DNA methylation levels across DE genes or random genes and flanking regions in WT and mutants. TSS and TTS indicate transcription start site and termination site, respectively. **d** Number of *kyp* hypoCHG DMRs and *cmt3a* hypoCHG DMRs within upstream 2 kb regions of DE genes or random genes. *Y*-axis represents the percentage of genes that have different numbers of DMRs in their upstream 2 kb regions. **e** Number of LTR TEs within upstream 2 kb regions of DE genes or random genes. *Y*-axis represents the percentage of genes that have different numbers of LTRs in their upstream 2 kb regions. T-tests are performed between DE genes and random genes and p-values are shown.

The *kyp* and *cmt3a* but not *cmt3b* mutants were stunted with wrinkled, asymmetric leaves with yellow spots, few and abnormal flowers, reduced fruit production and with no seeds (Fig. 1a). As DNA methylation may cause transcriptional silencing we predicted that the disrupted growth would be due to CHG hypomethylation in promoter regions leading to gene over-expression. Consistent with that idea, there were 362 upregulated genes and only 31 that were downregulated in *kyp* (Supplementary Data 1). Similarly, 340 genes were upregulated in *cmt3a* and 114 were downregulated (Supplementary Data 2). There is an overlap in half of the upregulated but very few of the downregulated genes in *kyp* and *cmt3a* (Fig. 1b).

Corresponding to the proposed link of DMRs with CMT- and KYP-mediated effects on gene expression, there was a dramatic reduction of CHG methylation and a slight reduction of CHH methylation in the gene body and flanking region of upregulated genes in *kyp* and *cmt3a* compared with random genes (Fig. 1c). Consistent with this pattern, there were more hypoCHG DMRs (both *kyp* DMRs and *cmt3a* DMRs) in upregulated genes in mutants compared with random genes (Fig. 1d).

It is likely that the CMT- and KYP-mediated DMRs and differentially expressed genes are related to TEs. The pericentric distribution of CMT-dependent CHG methylation and KYP-dependent CHG and CHH methylation (Supplementary Figs. 5d,e and S6), for example, coincides with the chromosomal distribution of Gypsy and Copia LTR elements identified by RepeatModeler^25^ and anti-correlates with the distribution of TIR elements and protein-coding genes (Supplementary Fig. 7). In addition, the overexpressed genes were more associated with LTR TEs than the random genes (Fig. 1e and Supplementary Fig. 8).

### KYP/CMT3a and RdDM in pericentric and distal chromatin

**Fig. 2:**
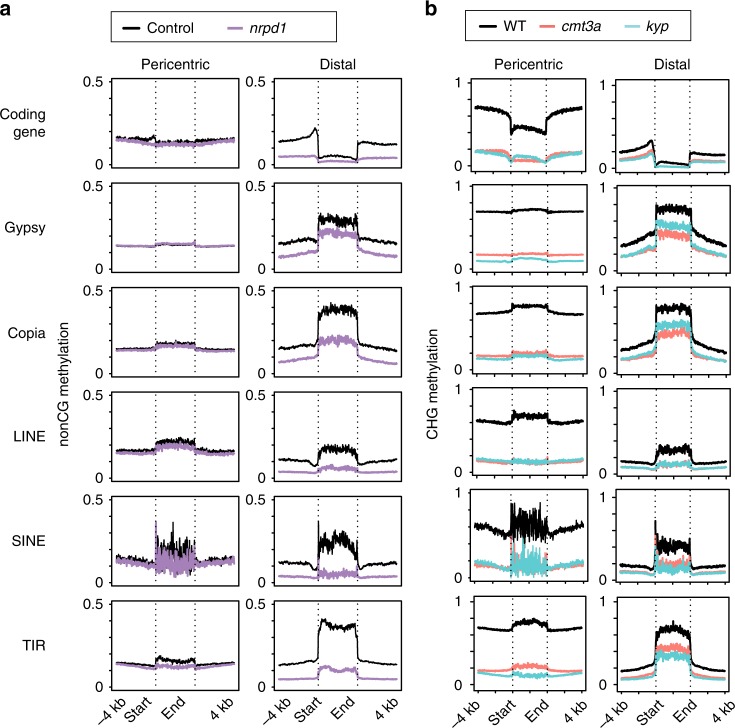
DNA methylation of TEs in pericentric and distal chromatin. **a** Average non-CG methylation over coding genes and different TE families in control and *nrpd1.* **b** Average CHG methylation in WT, *kyp* and *cmt3a*. Distal and pericentric chromatins are plotted separately. Different genomic elements are aligned at the 5′ end or the 3′ end, and average methylation for all cytosines within each 100 bp interval is plotted. The dotted lines represent the points of alignments of coding-gene transcriptional start site or annotated repeat elements start site and coding-gene transcription termination site or annotated repeat elements end site, respectively. TIR, terminal inverted repeats; LINE, long interspersed nuclear elements; SINE, short interspersed nuclear elements.

Therefore, to better understand CMT- and KYP-dependent CHG and CHH methylation, we analyzed the methylation pattern of TEs in the wild type and mutant plants. As the tomato chromosomes are predominantly pericentric heterochromatin we looked separately at the pericentric and distal chromosome regions (Fig. 2 and Supplementary Figs. 6 and 9). For comparison, we include data from an RdDM-defective *nrpd1* mutant that has a greater effect on CHH methylation in the distal chromosome arms than in the pericentric region^15^.

As in Arabidopsis^26^, the CHH methylation in distal TEs is primarily due to NRPD1 and KYP in pericentric regions (Supplementary Fig. 9a,b). At CHG NRPD1 has little effect (Supplementary Fig. 9c) but both KYP and CMT3a are influential at both pericentric and distal TEs (Fig. 2b). In the pericentric regions, however, the patterns of CMT3a- and KYP-mediated DNA methylation were not specific for TEs (Fig. 2b). There was a high level of CHG methylation and strong CHG hypomethylation both within these elements and also in the flanking DNA (Fig. 2b). The lack of specificity of *cmt3a* and *kyp* CHG hypomethylation in the pericentric region is reinforced by the strong CHG hypomethylation pattern at the transcribed and flanking region of protein-coding genes (Fig. 2b). This pattern is also consistent with the preferential pericentric distribution of upregulated genes in *cmt3a* and *kyp* (Supplementary Fig. 10).

In the distal chromatin, however, the TEs were specific foci of CHG and CHH methylation (Fig. 2a, b). NRPD1 mediates CHG and CHH methylation (non-CG methylation) and CMT3a and KYP mediates CHG methylation in the distal chromosomal domains (Fig. 2 and Supplementary Fig. 9) and the *cmt3a* and *kyp* CHG DMRs were less dense than in the pericentric region (Supplementary Fig. 5d, e). There were higher levels of CHG and CHH DNA methylation in the distal TEs than their flanking DNA and, with Gypsy, Copia and TIR class 2 type elements, there was a proximity effect: the CHG methylation in the flanking DNA decreased with increasing distance from the transposon (Fig. 2b). In all TEs there was CHG (or non-CG for *nrpd1*) hypomethylation in the mutants (Fig. 2a, b). In contrast there was very little differential DNA methylation in protein-coding genes in the distal chromatin (Fig. 2a, b). From these findings we conclude that there is a high degree of specificity of CMT3a, KYP and NRPD1 for transposons in the distal but not in the pericentric regions of tomato chromosomes (Fig. 2a, b).

### CMT3a effects anti-correlate RdDM at distal TEs

**Fig. 3:**
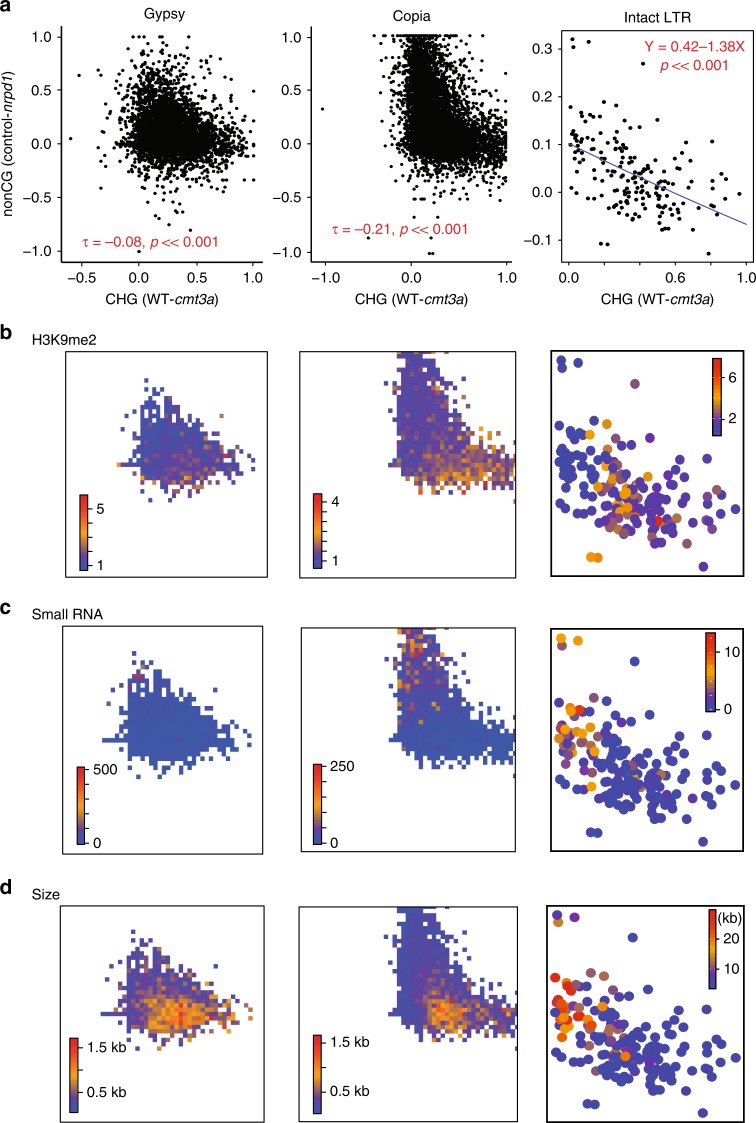
CMT3a-mediated CHG methylation anti-correlates NRPD1-mediated non-CG methylation in distal LTR TEs. **a** Scatter plots of hypomethylation of each TE. *X*-axis represents the reduction of CHG methylation in *cmt3a*, *y*-axis represents the reduction of non-CG methylation in *nrpd1*. Kendall’s tau test is performed for Gypsy and Copia. The Kendall rank correlation coefficients and p values relative to the null hypothesis of no correlation are indicated in red. Linear regression is performed for intact LTRs (the blue line). Regression model and the p-value relative to the null hypothesis that the line has zero gradient are indicated in red. **b**–**d** H3K9me2 (**b**), small RNA (**c**) and size (**d**) profiling of each TE. For Gypsy and Copia, color key represents average levels of H3K9me2 (**b**), small RNA (**c**) or size (**d**) of no less than three dots within each colored square. For intact LTR, color key represents levels of H3K9me2 (**b**), small RNA (**c**) or size (**d**) of each dot.

To investigate whether CMT3a-mediated CHG methylation coincides with NRPD1-mediated non-CG methylation in the distal chromatin (Fig. 2), we plotted the change in non-CG methylation due to *nrpd1* vs the change in *cmt3a-*mediated CHG methylation of each TE in each TE family (Fig. 3a and Supplementary Fig. 11a). With most element types (except LINE) there was an inverse relationship of non-CG hypomethylation in *nrpd1* with CHG hypomethylation in *cmt3a* (Fig. 3a and Supplementary Fig. 11a). This inverse relationship was most pronounced with the Gypsy, Copia and TIR elements (Fig. 3a and Supplementary Fig. 11a). The intact LTRs identified by LTRpred^27,28^ also show a similar negative correlation between non-CG hypomethylation in *nrpd1* and CHG hypomethylation in *cmt3a* (Fig. 3a).

Not surprisingly, given the biochemistry of plant epigenetics^2,3^, the distal *nrpd1*-hypomethylated elements were less associated with H3K9me2 and more associated with small RNA than the targets of CMT3a (Fig. 3b, c and Supplementary Fig. 11b,c). These trends were similar for distal Gypsy, Copia, TIR and for intact LTRs. With element size, however, there were opposite trends in distal Gypsy, Copia and TIR *vs* intact LTRs. With Gypsy, Copia and TIR the *nrpd1*-affected elements tended to be smaller (Fig. 3d and Supplementary Fig. 11d), as reported previously^24,26^. However, with the intact LTRs, the CMT3a targets were smaller (Fig. 3d).

To explain these data with the intact LTRs we invoke TE age. The intact LTRs targeted by RdDM tend to be very large (5–10 kb and above) which could reflect secondary transposition events into existing elements^29^. However, the smaller intact elements of approximately 5 kb may be close homologs of functional transposons that have recently mobilized. According to this age-based hypothesis the NRPD1 targeted intact LTRs in distal chromatin would be older than those affected by CMT3a (Fig. 3d).

### Preferential CHG methylation of younger LTRs by CMT3a

**Fig. 4:**
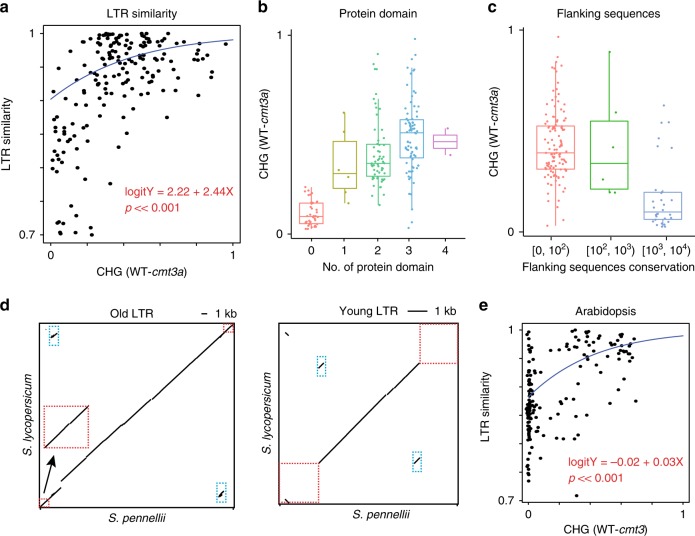
CMT3a-targeted intact LTRs are evolutionarily younger elements. **a** Scatter plot of distal intact LTRs. *X*-axis represents the reduction of CHG methylation in *cmt3a*, *y*-axis represents the 5′ and 3′ LTR similarity. Regression-blue line. The regression model and *p* value are indicated in red. The *p* value is relative to the null hypothesis of there being a gradient of zero and so no causal relationship between the reduction of CHG methylation in *cmt3a* and the 5′ and 3′ LTR similarity. **b** Correlation of number of predicted protein domains of each intact LTRs (*x*-axis) and reduction of CHG methylation in *cmt3a* (*y*-axis). **c** Alignments results of intact LTR flanking sequences (2 kb upstream and 2 kb downstream) between *S. lycopersicum* and *S. pennellii*. *X*-axis represents three ranges of the alignment score by BLASTn. *Y*-axis represents the reduction of CHG methylation in *cmt3a.* **d** Dot plots of each one example of old and young element comparing the LTR sequence from *S. lycopersicum* and its homologous sequence in *S. pennellii* genome, plus 2 kb upstream and downstream regions. Blue boxes highlight the 5′ and 3′ LTRs. Red boxes highlight the flanking regions. **e** Scatter plot of Arabidopsis distal intact LTRs. *X*-axis represents the reduction of CHG methylation in *cmt3*, *y*-axis represents the 5′ and 3′ LTR similarity. Regression-blue line. The regression model and p value are indicated in red. The p value is relative to the null hypothesis of there being a gradient of zero and so no causal relationship between the reduction of CHG methylation in *cmt3* and the 5′ and 3′ LTR similarity.

Three other features of the intact LTRs are consistent with this proposed age effect. First there was a positive correlation of the *cmt3a* hypoCHG methylation with 5′ and 3′LTR similarity that is a good indicator of LTR age^30^ (Fig. 4a). Second there is a correlation of *cmt3a* hypoCHG methylation with the presence of protein domains characteristic of transposition function (Fig. 4b). These protein domains are likely to be lost with transposon age. The third feature is in the flanking sequences of the intact LTRs that are more similar in *Solanum pennelli* and *Solanum lycopersicum* with the less *cmt3a* hypoCHG methylated elements (Fig. 4c, d). Conservation of flanking sequence is an indicator of older transposition events that occurred before the divergence of these two species.

This pattern of CMT-dependent silencing of younger LTRs in distal chromatin is characteristic of the Rider family of LTR retrotransposons in tomato^28^ (Supplementary Fig. 12) and it features with *Arabidopsis* LTRs*:* intact LTRs with CMT-dependent CHG methylation in *Arabidopsis* are larger and also have more similar LTRs than other elements (Supplementary Figs. 13 and 14 and Fig. 4e). CMT3a-mediated CHG methylation plays silencing roles on these young intact LTRs because the mRNA levels of these LTRs were upregulated in *cmt3a* than in WT (Supplementary Fig. 15).

## Discussion

**Fig. 5:**
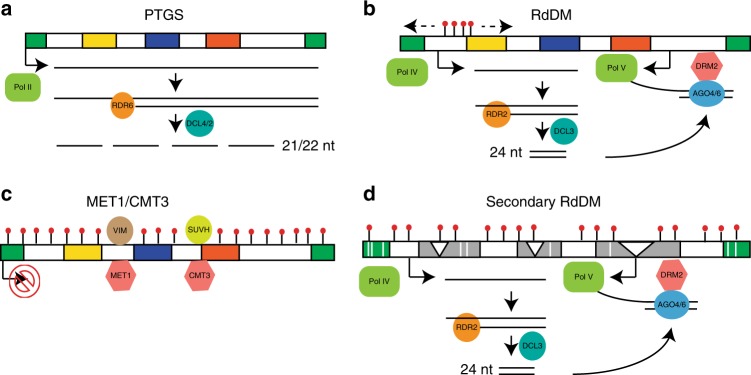
Model of LTR silencing. **a** In early stages, LTR is transcribed by RNA Pol II. Post-transcriptional gene silencing (PTGS) is triggered with double-stranded RNA synthesis by RDR6 and afterwards 21-22nt sRNA generation by DCL4 and/or DCL2. **b** Along with increase of LTR copy number, 24nt sRNA is produced starting from certain region of LTR (gag for example) by RNA Pol IV, RDR2 and DCL3. These 24nt sRNAs are recruited into AGO4/6 which interact with DNA methyltransferase DRM2. By sequence pairing between 24nt sRNA and Pol V transcript, AGO-DRM2 complex function *in cis* to methylate LTR which is called RNA-directed DNA methylation (RdDM). RdDM then spreads to more regions of LTR. **c** After established by RdDM, DNA methylation is maintained by RNA-independent mechanism: CG methylation is maintained by VIM and MET1, CHG methylation is maintained by KYP/SUVHs and CMT3. LTR is rarely transcribed. **d** Accumulated mutations in 5′ and 3′ LTRs and coding sequences (represented by white bars) together with TE re-insertion (represented by white triangles) make it less possible for LTR to transpose. At this stage, LTR is transcribed again and generates 24 nt sRNA, which mediates secondary RdDM.

Post-transcriptional gene silencing and RdDM had been previously implicated in retrotransposon silencing during the phases of retrotransposition and copy number increase^31^ (Fig. 5a, b). The RdDM elements are then thought to progress to RNA-independent silencing in which the CMT and MET1 methyltransferases would be involved^3,32^ (Fig. 5c). We interpret the KYP/CMT3a-dependent silencing of intact LTR elements (Figs. 3 and 4) as corresponding to that RNA-independent stage.

To account for our observations on H3K9me2, sRNA, transposon size and transposon age (Figs. 3 and 4) we propose that these elements would later undergo rearrangements and diminished transposon functionality. These rearranged elements would still be silenced by KYP/CMT3a (Fig. 5c) but, over time in the oldest elements, we envision that KYP/CMT3a-mediated CHG methylation would decrease and transcriptional silencing would be relaxed (Fig. 5d) so that RdDM would be re-established (Fig. 3).

According to this model there are separate phases of RdDM at two stages in the life and death cycle of retrotransposons. The first RdDM phase (Fig. 5b) would be short lived and observable only first few generations following invasion of a genome by a new transposon by introgression or horizontal gene transfer. This stage has been observed experimentally when the RNA-independent silencing of TEs has been released by mutation of *MET1, DDM1,* or *CMT3*^29,33,34^. It is likely that there are very few intact elements in wild type genomes undergoing this primary RdDM because RdDM mutants of *Arabidopsis* do not exhibit transposon mobilization unless other stresses are applied that might release CMT- and MET1-mediated silencing^35^.

A possible function of the sRNAs from the older elements in the second phase of RdDM is similar to that of piRNAs in animal systems: they would be a reservoir of *trans-*acting silencing factors that could protect against re-invasion of the genome by TEs with similar sequence identity^36^. They could also act *in trans* within the genome to influence the gene expression profile and the elements could also act *in cis* to affect expression of adjacent genes. The younger elements, in contrast, with CMT-dependent silencing would only have an effect in *cis* on adjacent genes. Clearly the progression between different modes of LTR silencing will have a profound influence of the ways that these TEs are controlling elements of gene expression in the host genome.

## Methods

### Plasmid construction

The CRISPR/Cas9 constructs were made following the Golden Gate cloning strategy^37^. A pair of sgRNAs were designed to target *KYP, CMT3a*, or *CMT3b* and amplified using pICH86966:AtU6p:sgRNA_PDS construct as a template. PCR products containing each sgRNA, together with pICSL01009:AtU6p providing the Arabidopsis U6 promoter, were cloned into level 1 constructs pICH47751 and pICH47761 respectively using BsaI (Thermo Fisher) and T4 DNA ligase (NEB). Together with other level 1 constructs (pICH47732:NOSp:NPTII, pICH47742:35S:Cas9) and the linker pICH41766, level 1 sgRNA constructs were assembled into the level 2 vector pAGM4723 using BpiI (Thermo Fisher) and T4 DNA ligase (NEB).

### Plant materials

All tomato plants used in this study are *Solanum lycopersicum* cv M82. *A. tumefaciens* strain AGL1 containing the CRISPR/Cas9 construct was used to transform tomato. In brief, tomato seeds were surfaced sterilized in 70% ethanol for 2 min followed by 2.2% sodium hypochlorite for 15 min and rinsed in sterilized water five times. The sterilized seeds were transferred to ½ strength Murashige-Skoog (MS) medium with vitamins, 0.8% agar, 1.5% sucrose. Cotyledons from 7-day-old plants were cut in two and submerged in a solution of Agrobacterium in MS liquid medium with 3% sucrose at OD600 = 0.5. The explants were then dried on filter paper and placed on a plate (1X MS medium, 0.6% agar, 0.5 mg/L 2,4-D) under dim light. After two days cultivation, the cotyledon segments were transferred to a selective regeneration medium (1X MS medium, 1X Nitsch vitamins, 0.1 g/L Myo inositol, 2% sucrose, 0.4% agar, 320 mg/L Timentin, 25 mg/L Cefotaxime, 2 mg/L Zeatin riboside, 100 mg/L Kanamycin). The later appearing shoots were transferred to a selective rooting medium (1X MS medium with vitamins, 2% sucrose, 2.25% gelrite, 320 mg/L Timentin, 50 mg/L Kanamycin). Regenerates with shoots and roots were then transferred into soil and genotyped. Primers for sgRNA amplification and mutant genotyping were listed in Supplementary Data 3.

### Bisulfite-seq

Genomic DNA was extracted from 100 mg of leaf tissue using Dneasy Plant Mini Kit (Qiagen). Bisulfite-seq library preparation was performed with three biological replicates for each genotype. 1 µg of genomic DNA was sonicated using the E220 Covaris instrument (Covaris Inc., USA) with parameters of incident power = 140 W, duty factor = 10%, cycles/burst = 200, treat time = 120 s. After being purified on XP beads (Ampure, ratio 1.8×), fragmented DNA was end-repaired and A-tailed using T4 DNA polymerase and Klenow Fragment (NEB) and purified again using XP beads (ratio 1.8×). Methylated Illumina Y-shaped adapters for paired-end sequencing were ligated using Quick-Stick ligase (Bioline). Purified (ratio 1×) adapter-ligated DNA was bisulfite-converted using the EZ DNA Methylation-Gold Kit (Zymo research). DNA was barcoded using 15 cycles of PCR amplification with KAPA HiFi HotStart Uracil+Ready Mix (KAPA Biosystems) with forward universal primer and reverse index primers. Pooled libraries were sequenced to a depth of about 12X genome coverage on a NextSeq 500 150PE (Illumina).

### Methylation analysis

Bisulfite-seq data generated in this study and Arabidopsis bisulfite-seq data generated by Stroud et al^24^ were used for further analysis. Tomato sequences from bisulfite-seq were trimmed and filtered by Trim Galore! with default parameters, and then mapped onto the tomato genome (Heinz SL3.0) using Bismark v0.15.0^38^ with option -N 1. Reads were deduplicated with deduplicate_bismark and methylation calls were extracted using bismark_methylation_extractor with option -ignore_r2 2. Raw data of Stroud et al^24^ (WT and *cmt3*) were downloaded from NCBI and processed following the same analyzing pipeline as tomato sequences, with exception of mapping onto the Arabidopsis genome (TAIR10). Differentially Methylated Region (DMR) analysis were performed with segmentSeq v3.8^39^. DMRs were called for each context separately, with cutoffs of width > 100 bp, FDR < 0.01 and likelihood > 0.99.

Average methylation profiles over genes and TEs were calculated from the cytosine reports with segmentSeq v3.8^39^ using the function of averageProfiles^39^. Annotations of tomato coding genes and TEs are based on SOL ITAG3.2. Arabidopsis and tomato intact LTRs were predicted by LTRpred (https://github.com/HajkD/LTRpred)^27,28^.

Methylation results of each TE were calculated from the cytosine reports with segmentSeq v3.8^39^ by averaging the methylation data of all cytosines of the same context within each TE.

### sRNA-seq

Total RNA was extracted by following the standard protocol of TRIzol method. 10 µg total RNA was run on a 15% PAGE/7 M Urea gel (Bio-Rad) and the sRNA fraction (18–25 nt) was excised and eluted from the gel, which was subsequently cloned using the NEBNext multiplex small RNA library prep kit (NEB). Libraries were indexed during the PCR step with 12 cycles and gel size-selected and purified. Four biological replicates of libraries for each genotype were constructed. Pooled libraries were sequenced on a NextSeq 500 (Illumina). Sequencing reads were trimmed using Trim Galore! with default parameters and then mapped to the Heinz genome SL3.0 using Bowtie 1.1.1^40^ with specified parameters of -m 1 and -v 0 for unique mapping and no mismatch allowed respectively. The output sam files were converted to bam files by Samtools^41^. The uniquely mapped bam files were used for RPKM analysis of sRNA.

### RNA-seq

Three micrograms of total RNA was purified using Ribo-Zero rRNA Removal Kit (Illumina). 250 ng purified RNA was used for RNA-seq library constructions following the manufacturer’s protocol of ScriptSeq RNA-Seq Library Kit (Illumina). RNA-seq libraries of three biological replicates for each genotype were sequenced on Illumina NextSeq 500. Sequencing reads were trimmed using Trim Galore! with default parameters and then mapped to the Heinz genome SL3.0 using Tophat v2.1.0^42^ with specified parameters of −N 0 and −r 330. The output sam files were converted to bam files by Samtools^41^ which were used for RPKM analysis.Differentially expressed genes were analyzed by DESeq2^43^ with cutoff of *p* < 0.01.

### ChIP-seq and distal/pericentric chromosome

ChIP-seq was performed following standard protocol^44^. Half gram of new leaves was ground in liquid nitrogen and in vitro cross-linked. The extracts were filtered through Miracloth and centrifuged for 20 min at 4000 rpm at 4 °C. The nuclei pellet was washed by extraction buffer (0.25 M sucrose, 10 mM Tris-HCl pH8.0, 10 mM MgCl_2_, 1 mM EDTA, 1% Triton X-100, 0.1 mM PMSF, 1 µM pepstatin, 1X protein inhibitor cocktails, 5 mM β-mercaptoethanol) and re-suspended in 0.6 ml of nuclear lysis buffer (50 mM Tris-HCl pH 8.0, 10 mM EDTA, 1% SDS, 0.1 mM PMSF, 1 µM pepstatin, 1X protein inhibitor cocktails) and sonicated using Bioruptor for 15 min (high, 30 s ON/30 s OFF). The lysate was centrifuged at 13,200 rpm for 10 min at 4 °C and the supernatant was kept as chromatin samples.

Antibodies of H3K9me2 (Abcam ab1220) and H3K9ac (Millipore 07-352) were used to perform ChIP on chromatin extracts. Libraries were constructed following manufacturer’s protocol of TruSeq Library Prep Kit (Illumina) and sequenced on Illumina NextSeq 500. Sequencing reads were trimmed using Trim Galore! with default parameters and then mapped to the Heinz genome SL3.0 using Bowtie2^40^. The output sam files were converted to bam files by Samtools^41^ which were used for RPKM analysis.

Normalized H3K9me2 and H3K9ac across each 100 kb window were calculated based on the ChIP-seq mapping files. Ratio of H3K9me2/H3K9ac was used to define distal/pericentric chromatin: H3K9me2/H3K9ac < 0.6, distal; H3K9me2/H3K9ac > 1.2, pericentric. Arabidopsis H3K9me2 ChIP-seq data^45^ was downloaded and processed following the same analysing pipeline as tomato sequences, with exception of mapping onto the Arabidopsis genome (TAIR10). The normalized H3K9me2 in each 100 kb window was calculated as the following formula:

H3K9me2 normalized level = Number of reads in each 100 kb window / (Number of total mapped reads/Number of 100 kb window in Arabidopsis genome)

### Reporting summary

Further information on research design is available in the Nature Research Reporting Summary linked to this article.

## Supplementary information

Supplementary Information

Peer Review File

Description of Additional Supplementary Files

Supplementary Data 1

Supplementary Data 2

Supplementary Data 3

Reporting Summary

## Data Availability

All raw sequencing data generated in this study have been deposited into in the NCBI Sequence Read Archive (SRA) under accession number PRJNA516166. The source data underlying Figs. 1a, 1c–e, 2a–b, 3a–d, 4a–c, 4e and Supplementary Figs 1c, 2e, 3a–c, 4a–c, 5d–e, 6, 7, 8, 9a–c, 10, 11a–d, 12, 13, 14, 15 are provided as Source Data file.

## Source Data

Source Data
