# Supplementary material for: Transposon age and non-CG methylation
Source: Nat Commun. 2020 Mar 6;11:1221. doi: 10.1038/s41467-020-14995-6 (PMC7060349; doi:10.1038/s41467-020-14995-6)
Supplement: Supplementary file 1 — Supplementary Information [file 41467_2020_14995_MOESM1_ESM.pdf]

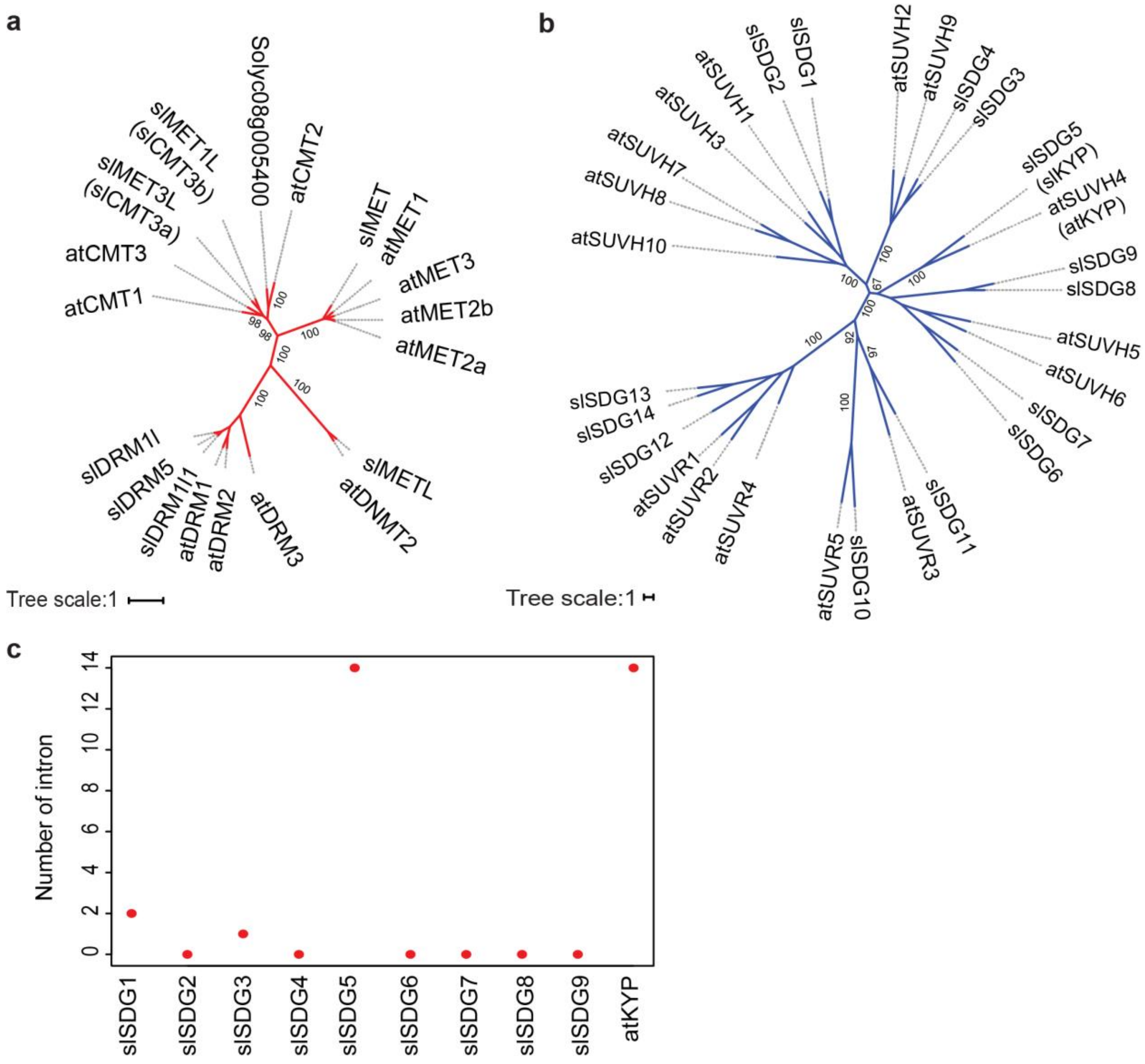

Supplementary Figure 1. Tomato orthologs of AtKYP and AtCMT3. (**a**-**b**) Phylogenetic tree of DNA methylases (**a**) and SUVHs and SUVRs (**b**) in tomato and Arabidopsis using amino acid sequences of each protein. Bootstrap values are labelled; (**c**) Intron number of tomato SUVH genes and Arabidopsis KYP gene.

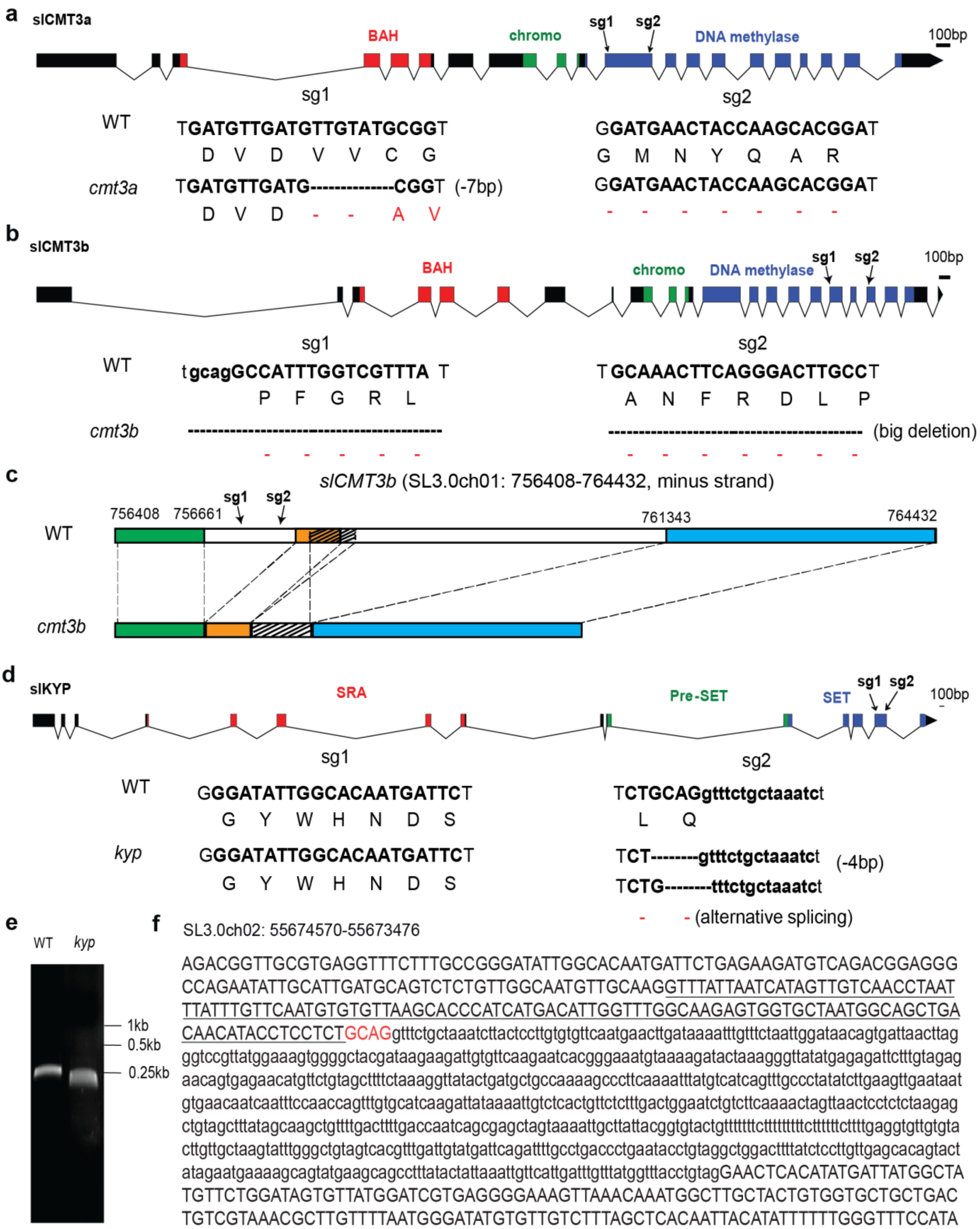

Supplementary Figure 2. Mutants in this study. Two T0 heterozygous mutants (one for each gene) were obtained and back crossed into WT M82 to reduce any off-target mutation by CRISPR. The later experiments used homozygous mutants in the selfed F2 lacking the Cas 9/gRNA construct with WT segregants as controls. (**a,b,d**) Gene structures of *slCMT3a* (**a**), *slCMT3b* (**b**) and *slKYP* (**d**). Exons and introns are indicated by rectangles and lines respectively. Functional domains are labelled with different colours. Small guide RNA (sgRNA) targets are marked with arrowheads. Encoded amino acids of sgRNA target regions are shown. Amino acid changes by mutation are highlighted in red. (**c**) Diagram of *CMT3b*

mutation. A 4,682bp deletion within *slCMT3b* (SL3.0ch01: 756661-761343) is found in *cmt3b.* There are two fragments (SL3.0ch01: 757590-757728 and SL3.0ch01: 757734-757618, inverted) inserted in this big deletion; (**e**) RT-PCR showing the alternative splicing of *slKYP* in *kyp*; (**f**) Alternative splicing of *slKYP* in *kyp*. Partial genomic sequence of *slKYP*. Exons and introns are indicated by uppercase and lowercase respectively. The “GCAG” marked in red stands for the 4bp deletion of DNA in *kyp*. Sequences with underline indicate the “new intron” in *kyp* caused by the 4bp deletion.

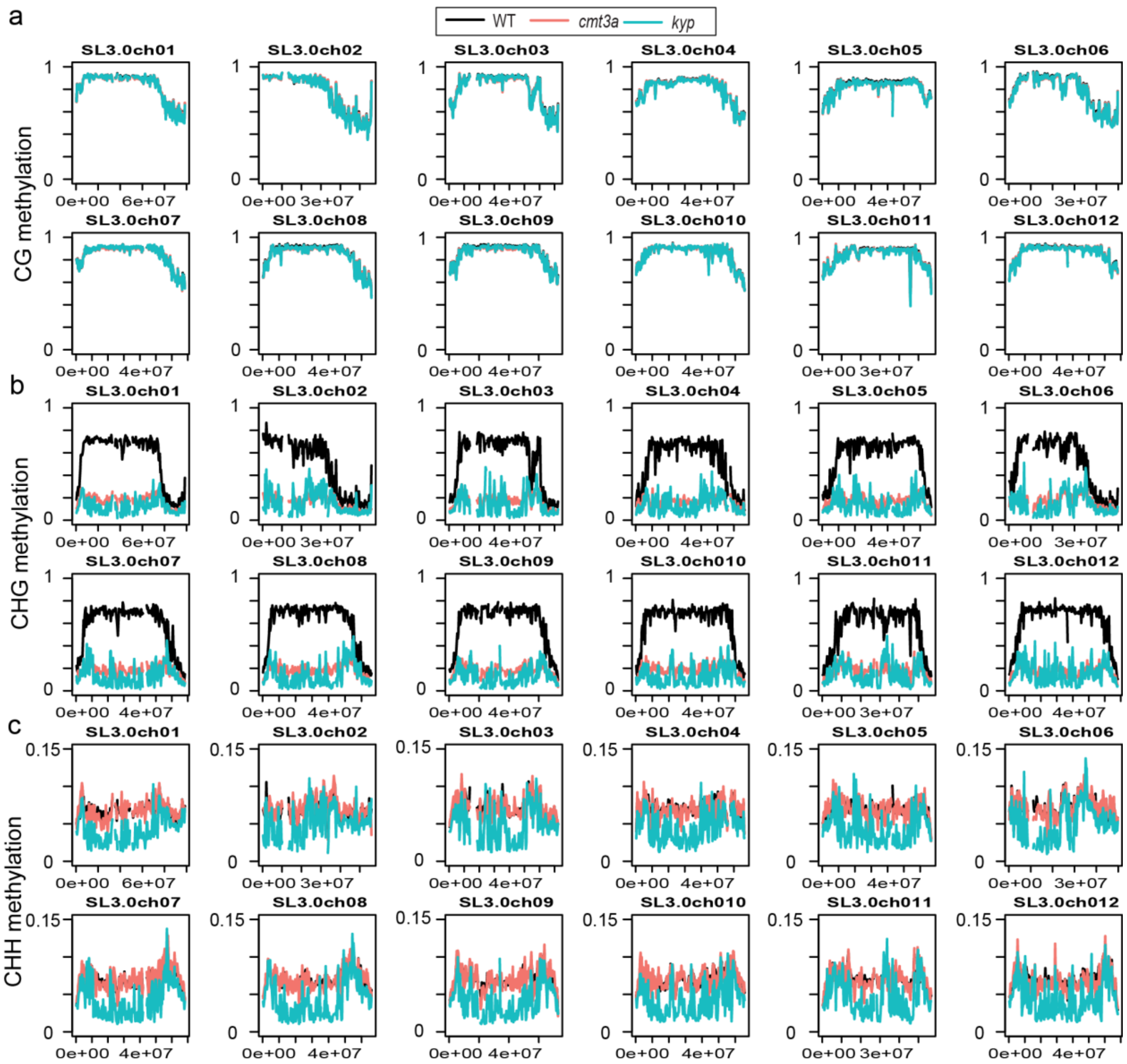

Supplementary Figure 3. DNA methylation levels of CG (**a**), CHG (**b**) and CHH (**c**) across 12 tomato chromosomes in leaves of WT, *kyp*, and *cmt3a*. X-axis represents coordinates of each chromosome, y-axis shows the methylation rate of each cytosine.

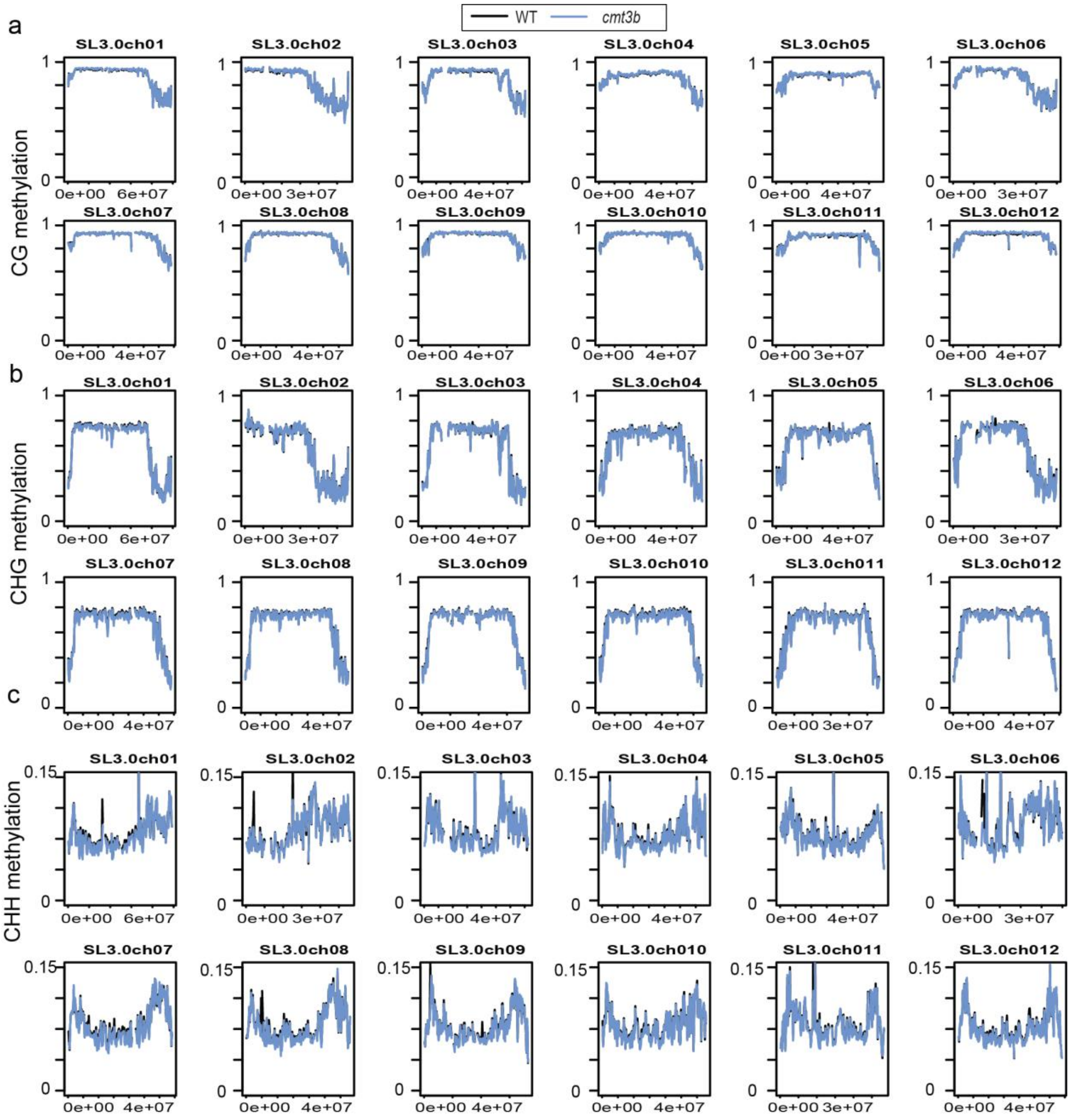

Supplementary Figure 4. DNA methylation levels of CG (**a**), CHG (**b**) and CHH (**c**) across 12 tomato chromosomes in leaves of WT and *cmt3b*. X-axis represents coordinates of each chromosome, y-axis shows the methylation rate of each cytosine.

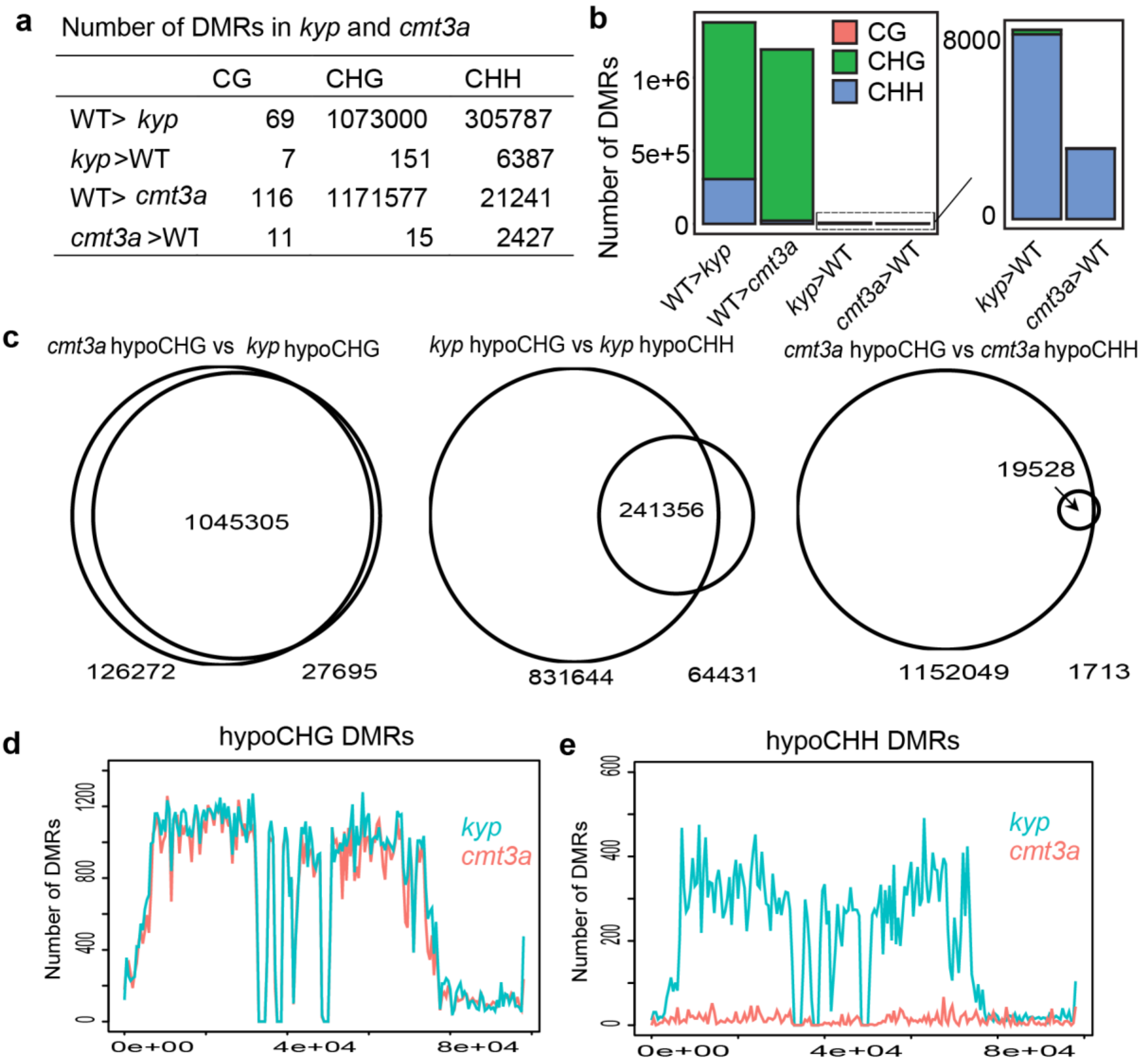
a Number of DMRs in *kyp* and *cmt3a*

	CG	CHG	CHH
WT> *kyp*	69	1073000	305787
kyp>WT	7	151	6387
WT> *cmt3a*	116	1171577	21241
cmt3a >WT	11	15	2427

Supplementary Figure 5. Differential methylated regions (DMRs). (**a**) Number of DMRs in *kyp* and *cmt3a;* (**b**) Boxplots of number of DMRs. Hypermethylation DMRs are zoomed in for better view. Besides hypoDMRs, there are fewer hyperDMRs (mostly CHH) in *kyp* and to a lesser extent *cmt3a* datasets, similarly to tomato *ddm1*[1]; (**c**) Venn diagrams showing overlap analysis of different DMRs; (**d-e**) Distribution of hypoCHG DMRs (**d**) and hypoCHH DMRs (**e**) across chromosome 1. Y-axis represents number of DMRs within each 500kb window.

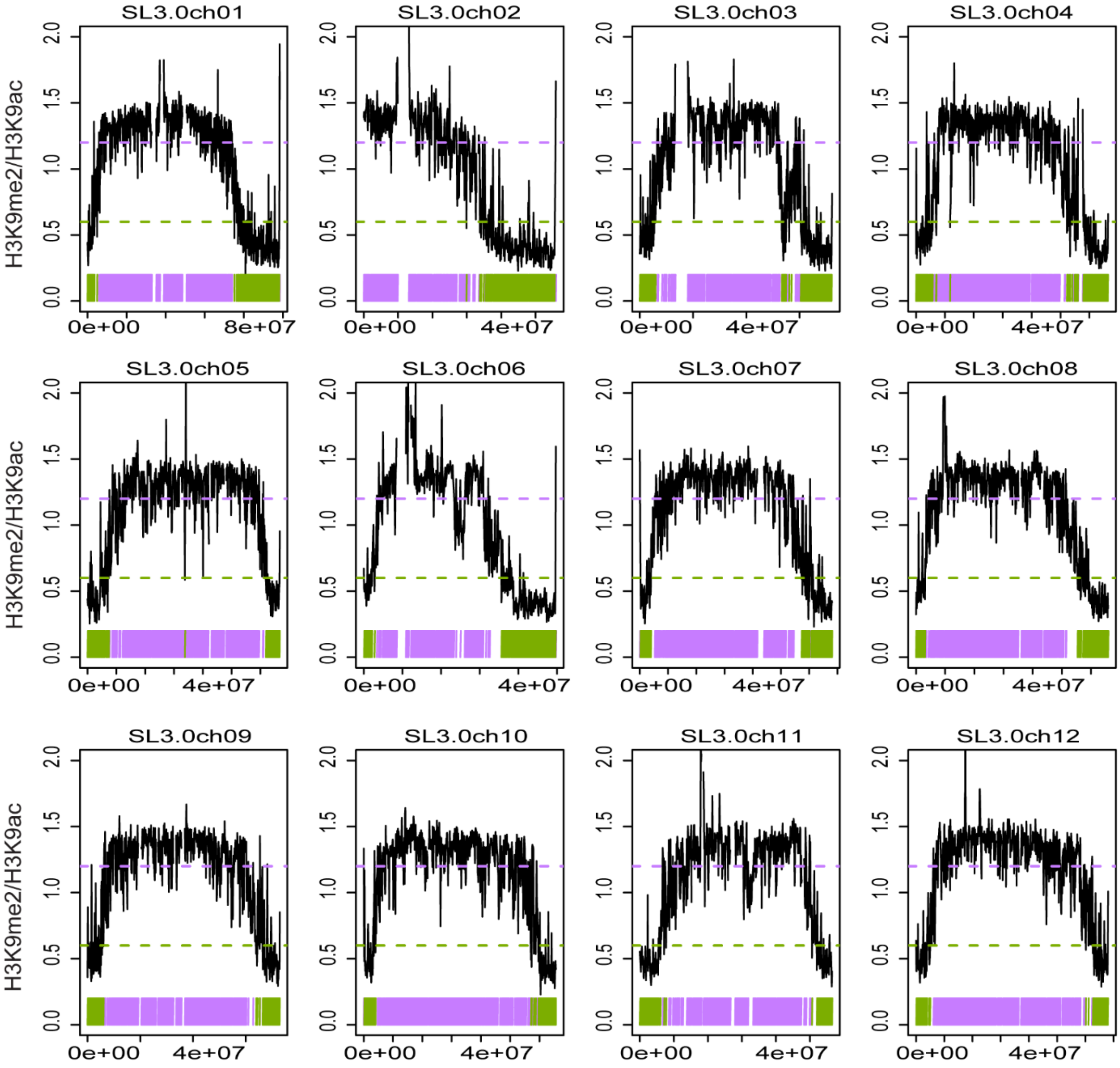

Supplementary Figure 6. Pericentric and distal chromatin are defined by ChIP-seq results of H3K9me2 and H3K9ac. Y-axis shows the ratio of normalized H3K9me2/H3K9ac within each 100kb window. Dash lines represent y=0.6 and y=1.2. Distal is defined as chromosomal regions with y<0.6, which is marked with green bars, whereas pericentric is defined as regions with y>1.2, which is marked with purple bars.

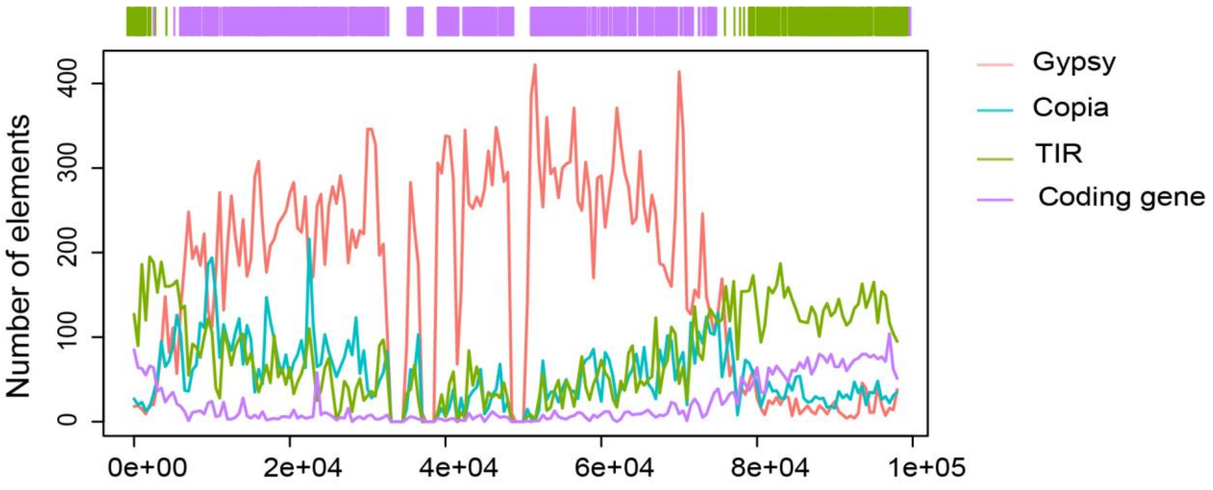

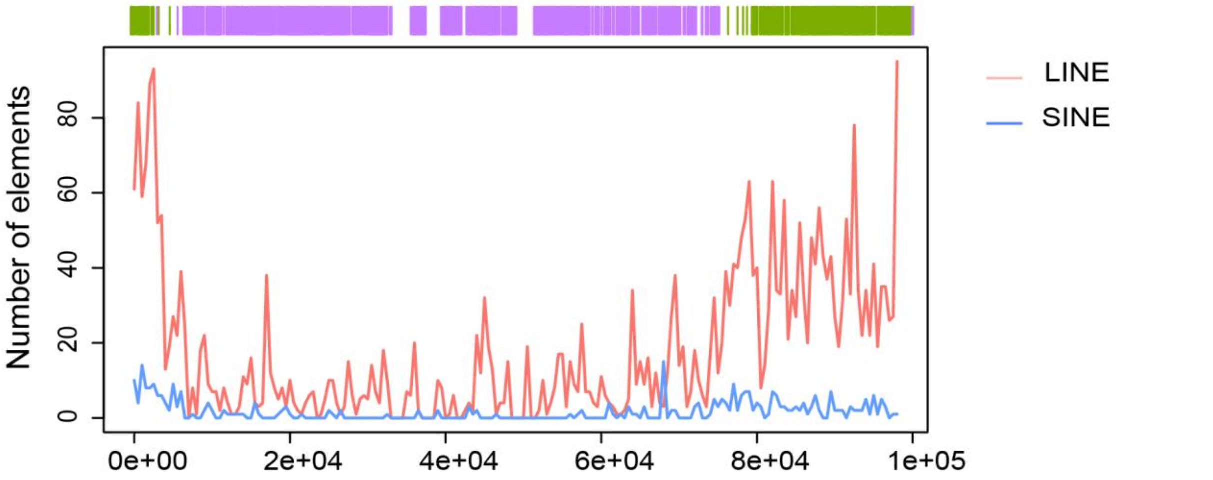

Supplementary Figure 7. Distribution of different genome features across chromosome 1. Y-axis represents number of different genome features within each 500kb window. Pericentric and distal chromatin are marked with purple and green bars respectively.

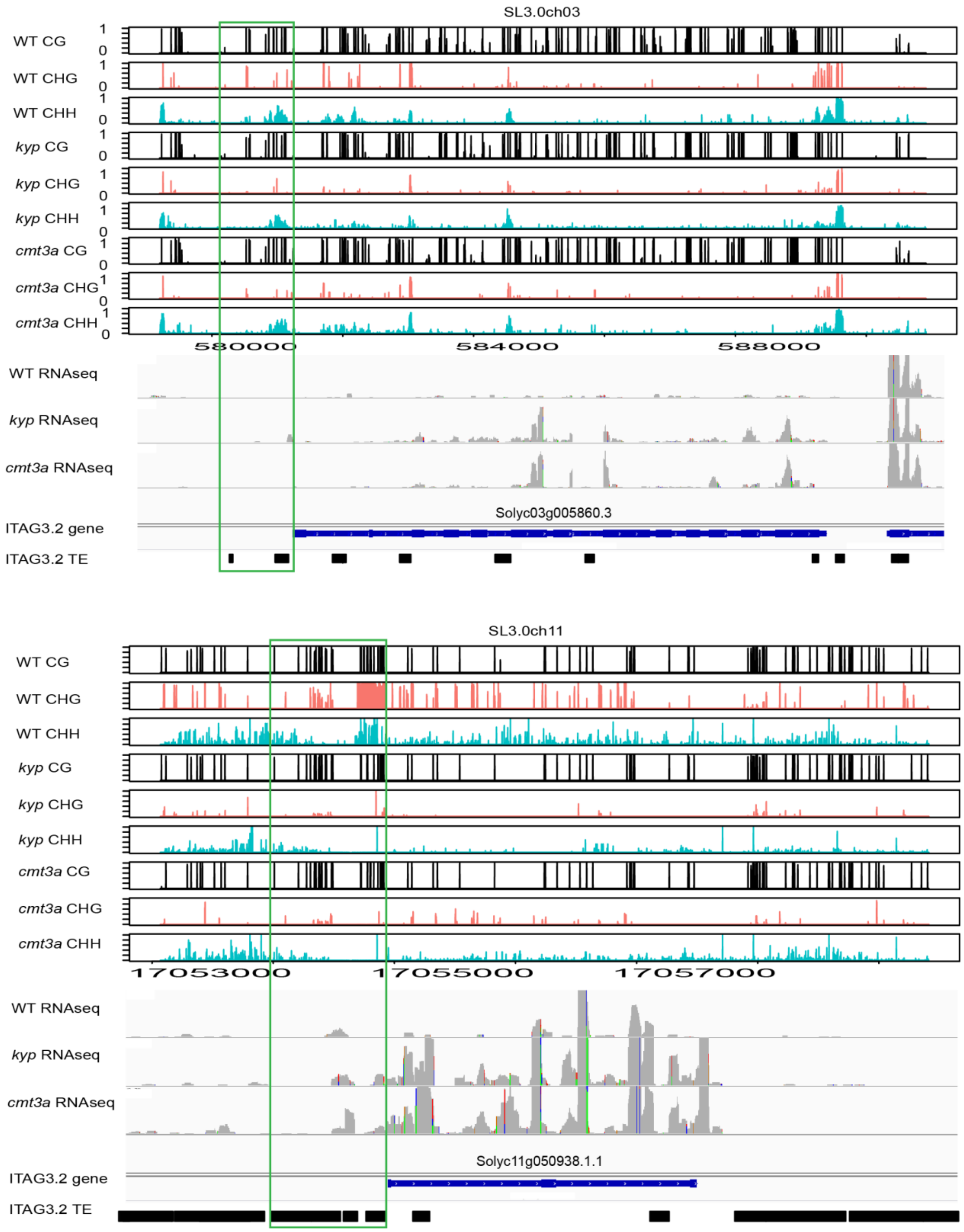

Supplementary Figure 8. Two examples of upregulated genes in *kyp* and *cmt3a*. Genome browser views of DNA methylation and normalized RNA-seq of each gene in WT and mutants. Annotated gene and TEs within this window are shown. Green boxes highlight the upstream TEs with hypomethylation in mutants, which might be "controlling elements".

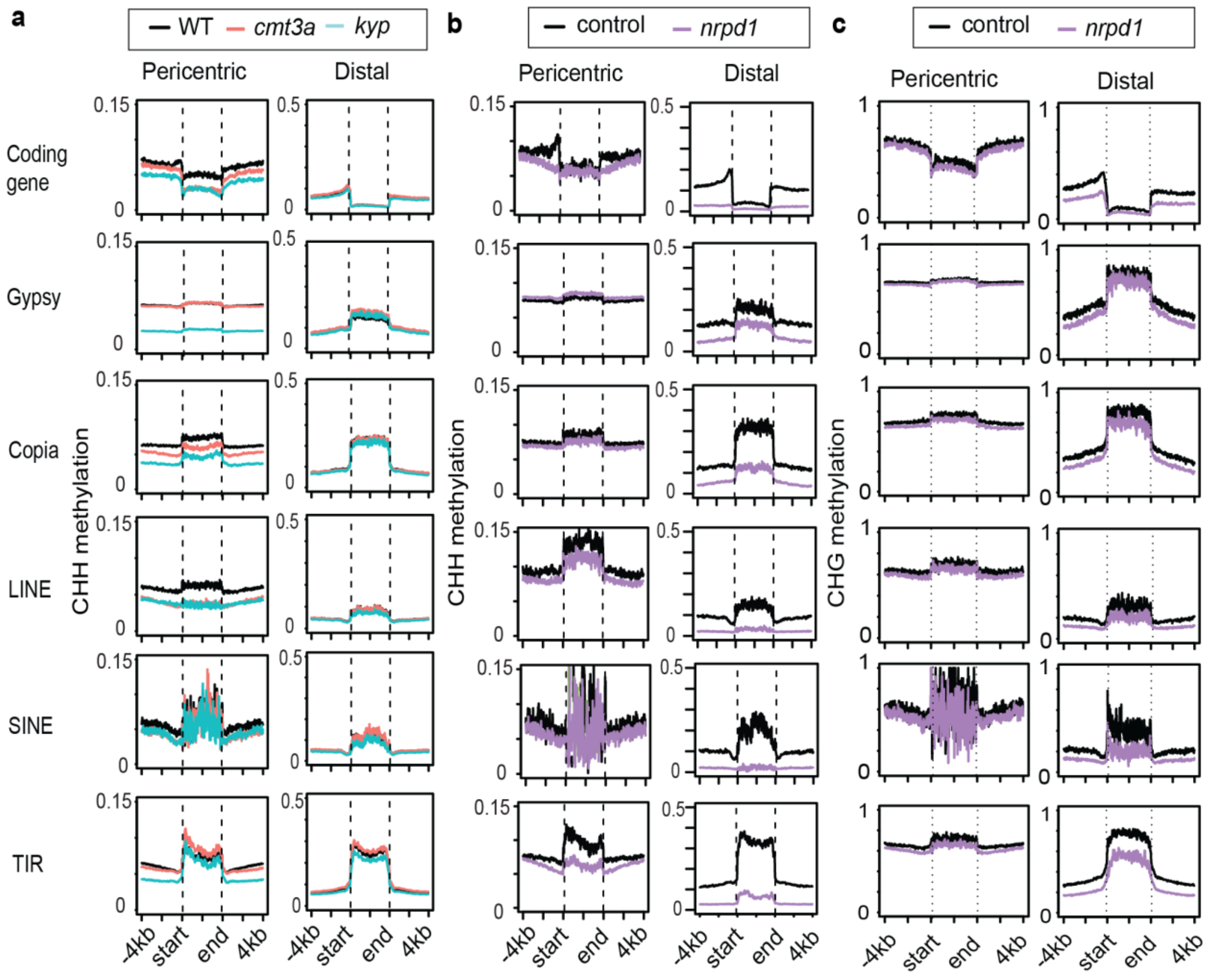

Supplementary Figure 9. (a-b) CHH methylation of TEs in pericentric and distal chromatin. Average CHH methylation over different TE families in WT, *kyp* and *cmt3a* (**a**) and control and *nrpd1* (**b**). **(c)** CHG methylation of TEs in pericentric and distal chromatin in control and *nrpd1*. Distal and pericentric chromatins are plotted separately. Different genomic elements are aligned at the 5' end or the 3' end, and average methylation for all cytosines within each 100bp interval is plotted. The dashed lines represent the points of alignments of coding-gene transcriptional start site or annotated repeat elements start site and coding-gene transcription termination site or annotated repeat elements end site, respectively. TIR, terminal inverted repeats; LINE, long interspersed nuclear elements; SINE, short interspersed nuclear elements.

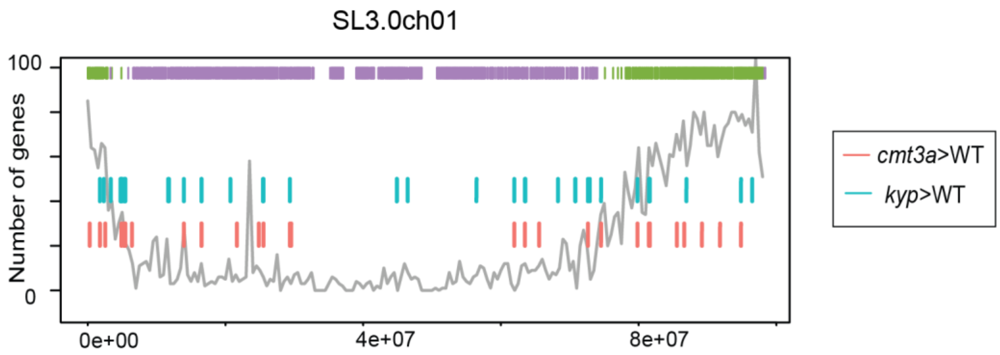

Supplementary Figure 10. Distribution of total genes and upregulated genes in *kyp* and *cmt3a* across chromosome 1. Y-axis represents number of total genes within each 500kb window. Vertical bars indicate locations of upregulated genes. Pericentric and distal chromatins are marked with purple and green bars respectively.

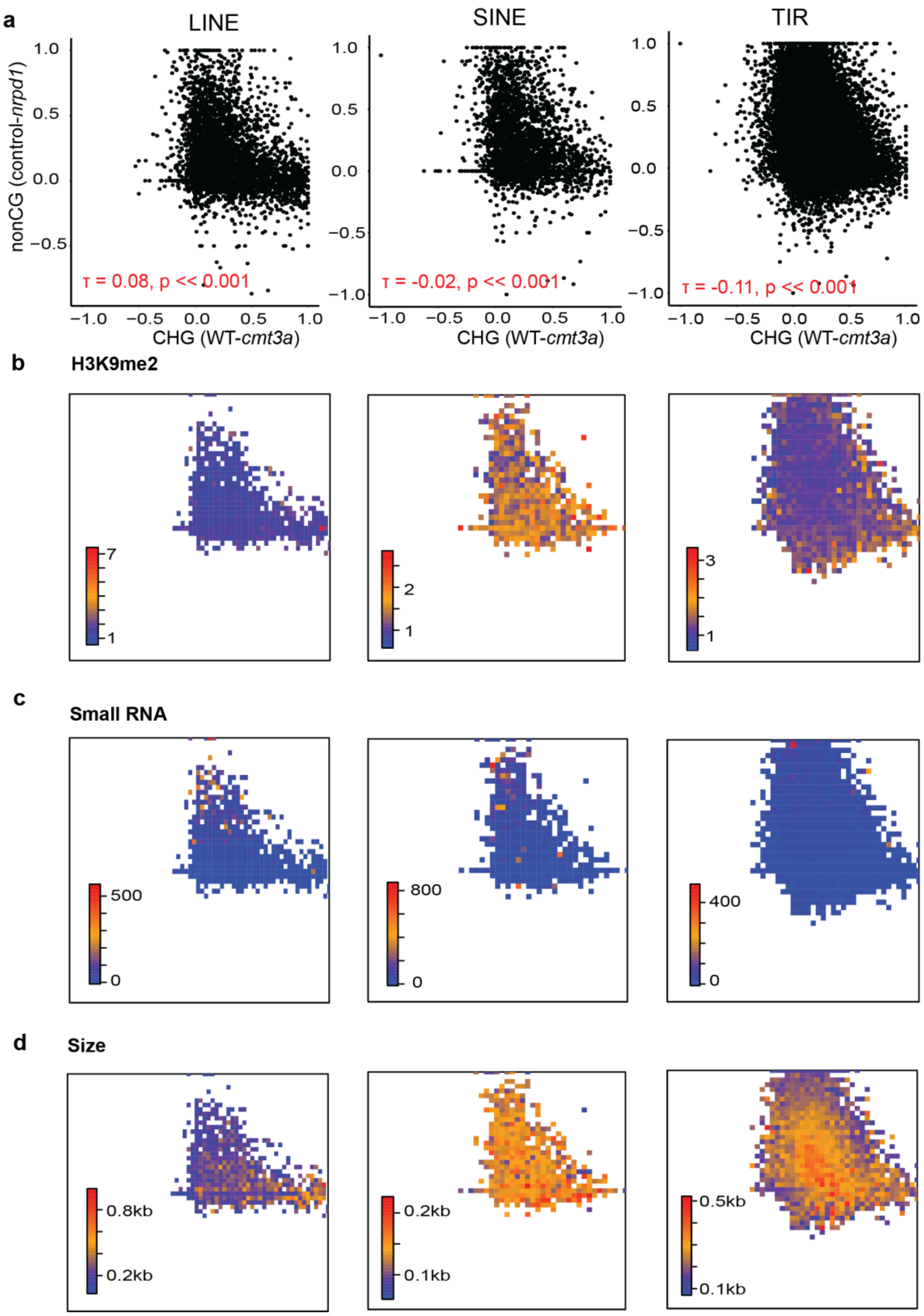

Supplementary Figure 11. (**a**) Scatter plots of hypomethylation of each TE. X-axis represents the reduction of CHG methylation in *cmt3a*, y-axis represents the reduction of non-CG methylation in *nrpd1*. Kendall's tau test is performed. The Kendall rank correlation coefficients and p values relative to the null hypothesis of no correlation are indicated in red; (**b-d**) H3K9me2 (**b**), small RNA (**c**) and size (**d**) profiling of TE. Colour key represents average levels of H3K9me2 (**b**), small RNA (**c**) or size (**d**) of no less than three dots within each coloured square.

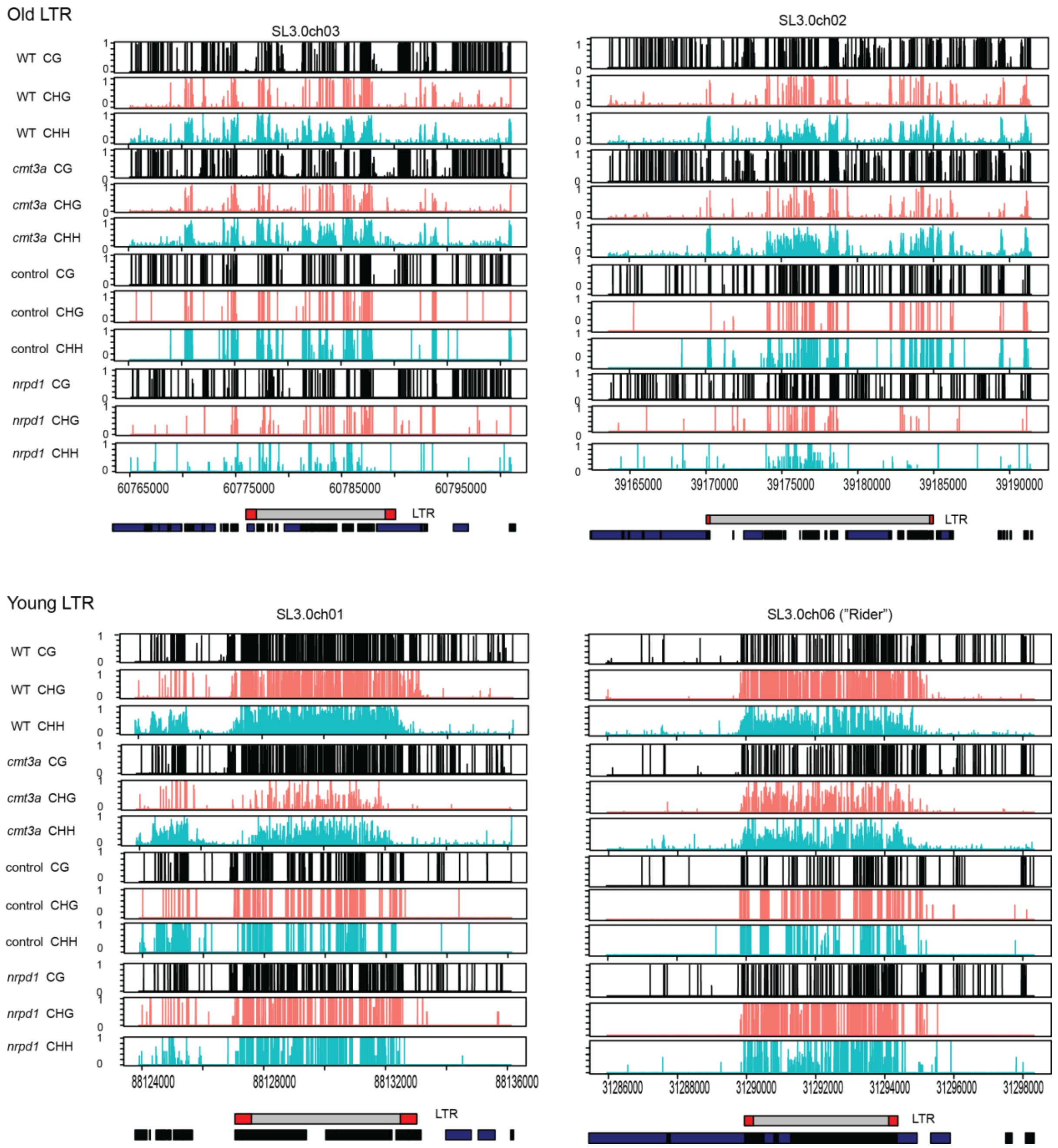

Supplementary Figure 12. Genome browser views of DNA methylation of four examples of intact LTRs in WT, *cmt3a*, control line and *nrpd1*. Grey bars stand for intact LTRs, in which red rectangles represent 5' and 3' LTRs. Blue and black bars indicate protein-coding genes and repetitive elements annotated in Sol ITAG3.2. One of the young LTRs is "Rider"[2].

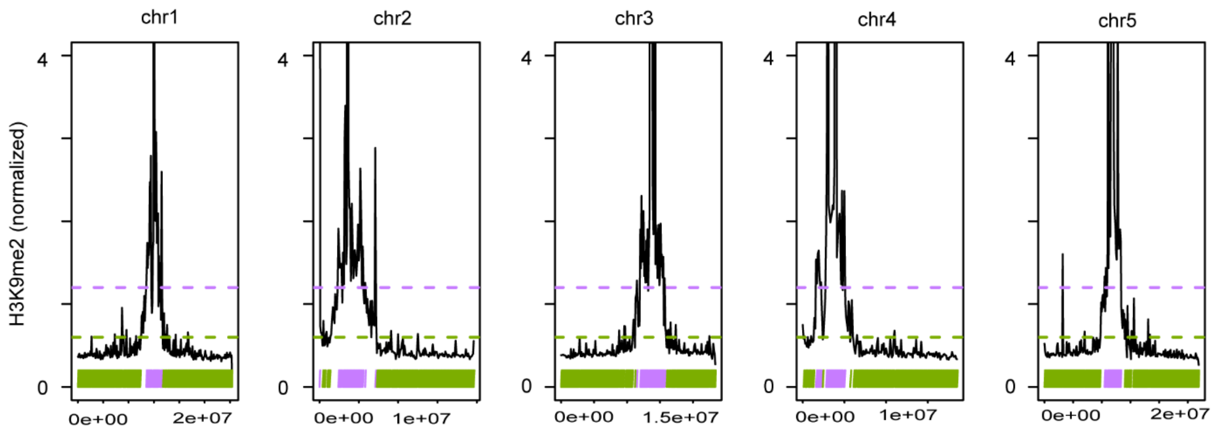

Supplementary Figure 13. Pericentric and distal regions in Arabidopsis identified by ChIP-seq results of H3K9me2. Y-axis shows the normalized H3K9me2 within each 100kb window. Dash lines represent y=0.6 and y=1.2. Distal chromatin is defined as chromosomal regions with y<0.6, which is marked with green bars, whereas pericentric heterochromatin is defined as regions with y>1.2, which is marked with purple bars.

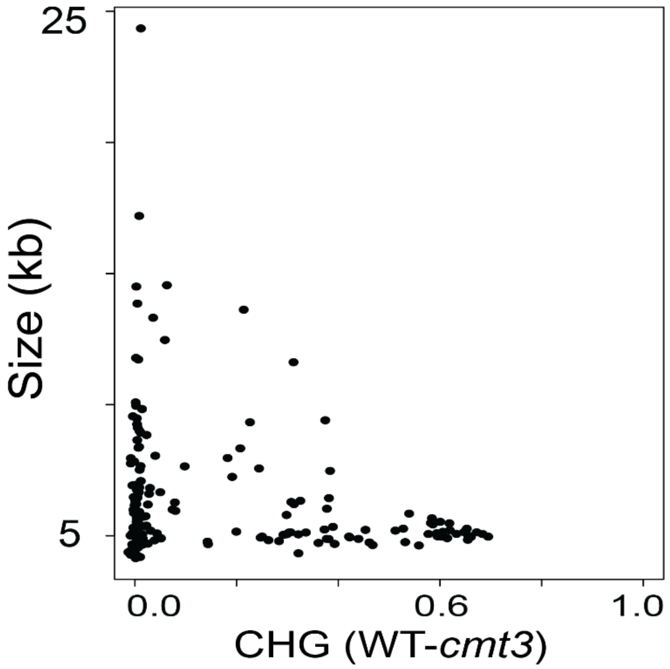

Supplementary Figure 14. Size profiling of Arabidopsis distal intact LTR. X-axis represents the reduction of CHG methylation in *cmt3*. y-axis represents the size of each LTR.

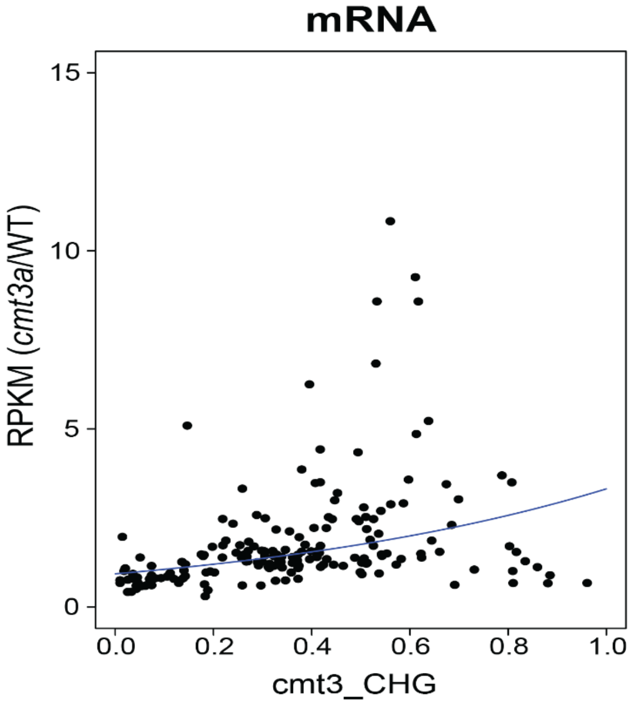

Supplementary Figure 15. Levels of mRNA of tomato distal intact LTR. X-axis represents the reduction of CHG methylation in *cmt3a*. y-axis represents the ratio of RPKM of each LTR in *cmt3a* to RPKM in WT. Regression-the blue line.

Supplementary References

1. Corem, S. *et al.* Redistribution of CHH Methylation and Small Interfering RNAs across the Genome of Tomato ddm1 Mutants. *Plant Cell* **30**, 1628–1644 (2018).

2. Benoit, M. *et al.* Environmental and epigenetic regulation of Rider retrotransposons in tomato. *PLoS Genet.* **15**, e1008370 (2019).
