# Supplementary material for: Transposon age and non-CG methylation
Source: Nat Commun. 2020 Mar 6;11:1221. doi: 10.1038/s41467-020-14995-6 (PMC7060349; doi:10.1038/s41467-020-14995-6)
Supplement: Supplementary file 3 — Description of Additional Supplementary Files [file 41467_2020_14995_MOESM3_ESM.pdf]

Description of Additional Supplementary Files

File Name: Supplementary Data 1
Description: Differential expressed genes between WT and kyp.

File Name: Supplementary Data 2
Description: Differential expressed genes between WT and cmt3a.

File Name: Supplementary Data 3
Description: Primers used in this study.
