# Supplementary material for: Transposon age and non-CG methylation
Source: Nat Commun. 2020 Mar 6;11:1221. doi: 10.1038/s41467-020-14995-6 (PMC7060349; doi:10.1038/s41467-020-14995-6)
Supplement: Supplementary file 7 — Reporting Summary [file 41467_2020_14995_MOESM7_ESM.pdf]

nature research

Corresponding author(s): David Baulcombe

Last updated by author(s): Jan 27, 2020

Reporting Summary

Nature Research wishes to improve the reproducibility of the work that we publish. This form provides structure for consistency and transparency in reporting. For further information on Nature Research policies, see Authors & Referees and the Editorial Policy Checklist .

Statistics

For all statistical analyses, confirm that the following items are present in the figure legend, table legend, main text, or Methods section.

n/a	Confirmed	
☐	☒	The exact sample size (*n*) for each experimental group/condition, given as a discrete number and unit of measurement
☐	☒	A statement on whether measurements were taken from distinct samples or whether the same sample was measured repeatedly
☐	☒	The statistical test(s) used AND whether they are one- or two-sided<br>*Only common tests should be described solely by name; describe more complex techniques in the Methods section.*
☒	☐	A description of all covariates tested
☐	☒	A description of any assumptions or corrections, such as tests of normality and adjustment for multiple comparisons
☐	☒	A full description of the statistical parameters including central tendency (e.g. means) or other basic estimates (e.g. regression coefficient) AND variation (e.g. standard deviation) or associated estimates of uncertainty (e.g. confidence intervals)
☐	☒	For null hypothesis testing, the test statistic (e.g. *F*, *t*, *r*) with confidence intervals, effect sizes, degrees of freedom and *P* value noted<br>*Give P values as exact values whenever suitable.*
☒	☐	For Bayesian analysis, information on the choice of priors and Markov chain Monte Carlo settings
☒	☐	For hierarchical and complex designs, identification of the appropriate level for tests and full reporting of outcomes
☒	☐	Estimates of effect sizes (e.g. Cohen's *d*, Pearson's *r*), indicating how they were calculated

Our web collection on statistics for biologists contains articles on many of the points above.

Software and code

Policy information about availability of computer code

Data collection	SRAtoolkit, v2.8.0<br>Illumina Basespace
Data analysis	Trim Galore, v0.4.1<br>TopHat2, v2.1.1<br>DESeq2, v1.24.0<br>Bowtie, v1.1.1<br>Bowtie2, v2.3.4.3<br>IGV, v2.3.20<br>Bismark, v0.20.0<br>Bedtools, v2.27.0<br>R, v3.6.0<br>Samtools, v1.9<br>segmentSeq, v3.8<br>LTRpred, available at https://github.com/HajkD/LTRpred

For manuscripts utilizing custom algorithms or software that are central to the research but not yet described in published literature, software must be made available to editors/reviewers. We strongly encourage code deposition in a community repository (e.g. GitHub). See the Nature Research guidelines for submitting code & software for further information.

Data

Policy information about availability of data

All manuscripts must include a data availability statement. This statement should provide the following information, where applicable:

- Accession codes, unique identifiers, or web links for publicly available datasets
- A list of figures that have associated raw data
- A description of any restrictions on data availability

Sequencing data generated in this study are deposited to NCBI SRA database as "PRJNA516166" and its identification is described in the Methods section.

Field-specific reporting

Please select the one below that is the best fit for your research. If you are not sure, read the appropriate sections before making your selection.

☒ Life sciences ☐ Behavioural & social sciences ☐ Ecological, evolutionary & environmental sciences

For a reference copy of the document with all sections, see nature.com/documents/nr-reporting-summary-flat.pdf

Life sciences study design

All studies must disclose on these points even when the disclosure is negative.

Sample size	No sample-size calculation was performed. Samples size was chosen according to the standard generally accepted in the field of plant molecular biology.
Data exclusions	No data were excluded from the analyses.
Replication	All the experimental findings were reproducible in the independent biological replications.
Randomization	Plants were placed randomly in the growing facility.
Blinding	No blinding was applied. Most of the data in this study are generated by bioinformatic analyses. Since we applied identical settings to all the samples, blinding was not essential.

Reporting for specific materials, systems and methods

We require information from authors about some types of materials, experimental systems and methods used in many studies. Here, indicate whether each material, system or method listed is relevant to your study. If you are not sure if a list item applies to your research, read the appropriate section before selecting a response.

Materials & experimental systems

n/a	Involved in the study	
☐	☒	Antibodies
☒	☐	Eukaryotic cell lines
☒	☐	Palaeontology
☒	☐	Animals and other organisms
☒	☐	Human research participants
☒	☐	Clinical data

Methods

n/a	Involved in the study	
☐	☒	ChIP-seq
☒	☐	Flow cytometry
☒	☐	MRI-based neuroimaging

Antibodies

Antibodies used	anti-H3K9me2, abcam ab1220; anti-H3K9ac, Millipore 07-352
Validation	Validation statements of both antibodies are available from manufactures: H3K9me2 (https://www.abcam.com/histone-h3-di-methyl-k9-antibody-mabcam-1220-chip-grade-ab1220.html) and H3K9ac (https://www.merckmillipore.com/GB/en/product/Anti-acetyl-Histone-H3-Lys9-Antibody,MM_NF-07-352?ReferrerURL=https%3A%2F%2Fwww.google.com%2F&bd=1).

ChIP-seq

Data deposition

- [x] Confirm that both raw and final processed data have been deposited in a public database such as GEO.
- [ ] Confirm that you have deposited or provided access to graph files (e.g. BED files) for the called peaks.

Data access links<br>*May remain private before publication.*	All the data has been deposited to the NCBI SRA database (PRJNA516166).
Files in database submission	Files in database submission will be provided in supplementary tables.
Genome browser session<br>(e.g. UCSC)	N.A.

Methodology

Replicates	Two replicates of each experiment were performed.
Sequencing depth	Sequencing depth information will be provided in supplementary tables.
Antibodies	anti-H3K9me2, abcam ab1220; anti-H3K9ac, Millipore 07-352
Peak calling parameters	No peak calling was performed. ChIP-seq data were used to define the distal and pericentric chromatins.
Data quality	Sequencing data quality was accessed by FastQC (https://www.bioinformatics.babraham.ac.uk/projects/fastqc/).
Software	Trim Galore, v0.4.1<br>Bowtie2, v2.3.4.3
