# Supplementary material for: Transposon age and non-CG methylation
Source: Nat Commun. 2020 Mar 6;11:1221. doi: 10.1038/s41467-020-14995-6 (PMC7060349; doi:10.1038/s41467-020-14995-6)
Supplement: Supplementary file 2 — Peer Review File [file 41467_2020_14995_MOESM2_ESM.pdf]

Editorial Note: This manuscript has been previously reviewed at another journal that is not operating a transparent peer review scheme. This document only contains reviewer comments and rebuttal letters for versions considered at Nature Communications.
